# Quantum spinning photonic circulator

**DOI:** 10.1038/s41598-022-09626-7

**Published:** 2022-04-07

**Authors:** Yu-Wei Jing

**Affiliations:** Key Laboratory of Low-Dimensional Quantum Structures and Quantum Control of Ministry of Education, Key Laboratory for Matter Microstructure and Function of Hunan Province, Department of Physics and Synergetic Innovation Center for Quantum Effects and Applications, Hunan Normal University, Changsha, 410081 China

**Keywords:** Optical materials and structures, Optical physics, Single photons and quantum effects

## Abstract

We propose a scheme to realize a four-port quantum optical circulator for critical coupling of a spinning Kerr resonator to two tapered fibers. Its nonreciprocal effect arises from the Fizeau drag induced splitting of the resonance frequencies of the two counter-travelling optical modes. The transmitted photons exhibit direction dependent quantum correlations and nonreciprocal photon blockade occurs for photons transferred between the two fibers. Moreover, the quantum optical circulator is robust against the back scattering induced by intermodal coupling between counter-travelling optical modes. The present quantum optical circulator has significant potential as an elementary cell in chiral quantum information processing without magnetic field.

## Introduction

Nonreciprocal optical devices, such as circulators and isolators, which feature direction dependent photons propagation, are fundamental elements for signals routing and processing in photonic circuits^1–6^. The key requirements to achieve nonreciprocity is breaking the time-reversal symmetry and Lorentz reciprocity in linear and nonmagnetic media^7,8^. Besides the traditional nonreciprocal devices with magnetic materials^9–11^, many magnetic-free systems with broken time-reversal symmetry have been proposed theoretically and demonstrated experimentally in the past few years, such as Kerr-nonlinear microresonators^12–16^, optomechanical systems^17–37^, non-Hermitian systems^38–43^, moving atomic gases^44–52^, and spinning resonators^53–55^.

In a recent experiment^53^, an optical isolator with 99.6% isolation was demonstrated by using a tapered fiber coupling a spinning resonator. The nonreciprocal effect for this optical isolator arises from the Fizeau drag induced splitting of the resonance frequencies of the counter-travelling optical modes in the spinning resonator, which breaks the time-reversal symmetry of the system. A spinning resonator with broken time-reversal symmetry provides an ideal platform for the realization of nonreciprocal devices^53^, and for enhancing the optical sensing of nanoparticle with single-particles resolution^54^. Moreover, based on this platform, several new nonreciprocal effects were predicted, such as nonreciprocal photon blockade^55–60^, nonreciprocal phonon laser^61,62^, nonreciprocal entanglement^63^, and nonreciprocal optical solitons^64^. However, until recently, there is no literature on the subject of optical circulator based on a spinning resonator.

As one of the most common nonreciprocal optical devices, circulator is an indispensable optical device and, in a sense, even more irreplaceable than isolator. Because a isolator can be realized by just using two ports of a circulator, but a circulator cannot be replaced by combining isolators. The design of circulators for electromagnetic fields have achieved significant progress during the past decade. Circulators have been demonstrated experimentally for microwave^65–68^ and optical^69–71^ signals, and can be applied from classical to quantum regime^71–74^.

In this paper, we are going to propose a scheme to realize a four-port quantum optical circulator for critical coupling of a spinning Kerr resonator to two tapered fibers. Different from the previous studies^53–59^ based on spinning resonators only limited to two-port devices, we focus on a circulator with four ports and show that the circulator routes the incident photons from one input port to the adjacent output port clockwise or counterclockwise, dependent on the frequency of the incident photons and the rotating direction of the spinning resonator. Interestingly, the transmitted photons also exhibit direction dependent quantum correlations, so that the optical circulator can be used for quantum optical applications. In addition, quantum optical circulator based on a spinning Kerr resonator has several advantages over its counterparts, such as robust against the back scattering^63,64^.

## Results

### Physical model

**Figure 1:**
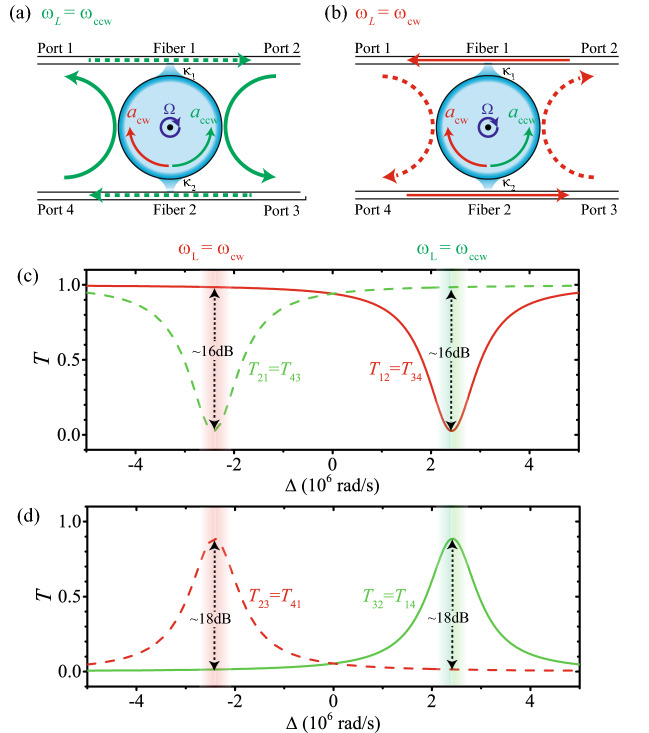
(Color online) Routing behaviors of the circulator for the input field with frequency (**a**) $\omega _{L}=\omega _\mathrm{ccw}= \omega _\mathrm{c}+\Delta _F$ and (**b**) $\omega _{L}=\omega _\mathrm{cw}= \omega _\mathrm{c}-\Delta _F$. (**c**) and (percentisolationwasdemonstratedbyu) Port-to-port transmission spectra versus the detuning $\Delta$.

We consider a spinning Kerr resonator, with clockwise (CW) and counter-clockwise (CCW) travelling modes $a_\mathrm{cw}$ and $a_\mathrm{ccw}$, coupling to two tapered fibers with four input/output ports, as shown in Fig. 1a,b. For the Kerr resonator with stationary resonance frequency $\omega _c$ spinning clockwise (top view) at an angular velocity $\Omega$, the effective frequency for the CW and CCW travelling modes are $\omega _\mathrm{cw}=\omega _c - \Delta _{F}$ and $\omega _\mathrm{ccw}=\omega _c + \Delta _{F}$, with rotation induced Sagnac-Fizeau shift^75^

1 $$\begin{aligned} \Delta _{F}=\frac{nR\Omega \omega _c}{c}\left( 1-\frac{1}{n^2}-\frac{\lambda }{n}\frac{dn}{d\lambda }\right) . \end{aligned}$$

Here *c* and $\lambda$ are the speed and wavelength of light in vacuum, *n* and *R* are the refractive index and radius of the resonator, respectively. In a typical material for microtoroid resonator (e.g. silica^76^), the dispersion term $dn/d\lambda$ originates from the relativistic correction of the Sagnac effect is relatively small ($< 1\%$) and can be ignored safely^53,75^.

In a frame rotating at driving frequency $\omega _L$, the system can be described by the Hamiltonian as ($\hbar =1$)

2 $$\begin{aligned} H_{\mathrm {sys}}=H_{0}+H_{\mathrm {dri}}, \end{aligned}$$

where $H_0$ is the Hamiltonian of the spinning Kerr resonator

3 $$\begin{aligned} H_{0} = (- \Delta - \Delta _{F}) a_{\mathrm {cw}}^{\dag }a_{\mathrm {cw}} + U a_{\mathrm {cw}}^{\dag }a_{\mathrm {cw}}^{\dag }a_{\mathrm {cw}}a_{\mathrm {cw}} +(- \Delta + \Delta _{F}) a_{\mathrm {ccw}}^{\dag }a_{\mathrm {ccw}} + U a_{\mathrm {ccw}}^{\dag }a_{\mathrm {ccw}}^{\dag }a_{\mathrm {ccw}}a_{\mathrm {ccw}} , \end{aligned}$$

with detuning $\Delta \equiv \omega _L-\omega _c$ and nonlinear interaction strength *U*, and $H_\mathrm{dri}$ given by

4 $$\begin{aligned} H_{\mathrm {dri}}=-i \sqrt{\kappa _{\mu }}\alpha ^{*}a_{\mathrm {\nu }} +i \sqrt{ \kappa _{\mu }}\alpha a_{\nu }^{\dag }, \end{aligned}$$

denotes coherent driving of the CW or CCW travelling mode (subscript $\nu =\mathrm{cw}$ or $\mathrm ccw$) from the tapered fiber $\mu$ ($\mu =1$ or 2) with coupling rates $\kappa _{\mu }$. The system are adjusted so that both fibers are approximately critically coupled to the resonator: $\kappa _{1}=\kappa _{2}=\kappa \gg \kappa _{0}$, where $\kappa _{0}$ is the intrinsic decay rate of the CW and CCW travelling modes. $\alpha =\sqrt{P_\mathrm{in}/(\hbar \omega _L)}$ is the driving strength with driving power $P_\mathrm{in}$. The Kerr-type nonlinear interaction strength is given by $U=\hbar \omega _c^2 c n_2 /(n_0^2 V_\mathrm{eff} )$, where $n_0$ and $n_2$ are the linear and nonlinear refraction indexes, and $V_\mathrm{eff}$ is the effective mode volume.

According to the input-output relations^77^, the output field from the four ports are given by

5 $$\begin{aligned} a_{1,\mathrm {out}}&=\sqrt{\kappa _{1}}a_{\mathrm {ccw}}-a_{2,\mathrm {in}}, \end{aligned}$$

6 $$\begin{aligned} a_{2,\mathrm {out}}&=\sqrt{\kappa _{1}}a_{\mathrm {cw}}-a_{1,\mathrm {in}}, \end{aligned}$$

7 $$\begin{aligned} a_{3,\mathrm {out}}&=\sqrt{\kappa _{2}}a_{\mathrm {ccw}}-a_{4,\mathrm {in}}, \end{aligned}$$

8 $$\begin{aligned} a_{4,\mathrm {out}}&=\sqrt{\kappa _{2}}a_{\mathrm {cw}}-a_{3,\mathrm {in}} \end{aligned}$$

with coherent driven field or vacuum field input from port *i* ($i=1,2,3,4$) given by $a_{i,\mathrm {in}}$. To quantify the performance of the circulator, we will calculate the transmission coefficient for photon from port *i* to port *j* by

9 $$\begin{aligned} T_{ji}\equiv \frac{\left\langle a_{j,\mathrm {out}}^{\dag }a_{j,\mathrm {out} }\right\rangle }{\left\langle a_{i,\mathrm {in}}^{\dag }a_{i,\mathrm {in} }\right\rangle }, \end{aligned}$$

and the statistic properties of the transmitted photons can be described by the equal-time second-order correlation function in the steady state ($t\rightarrow \infty$) as

10 $$\begin{aligned} g_{ji}^{\left( 2\right) }\left( 0 \right) \equiv \left. \frac{\left\langle (a_{j,\mathrm {out}}^{\dag })^2 (a_{j,\mathrm {out}})^2 \right\rangle }{\left\langle a_{j,\mathrm {out}}^{\dag } a_{j,\mathrm {out}} \right\rangle ^{2}} \right| _{j \leftarrow i}, \end{aligned}$$

where $j \leftarrow i$ denotes that the transmitted photons are coming from port i. In the following we will obtain the transmission and statistical properties of the photons quantitatively by solving the master equation given in the Methods numerically, with the experimentally accessible parameters^76,78,79^ as: $R=30\,\mu$m, $Q\equiv \omega _c/\kappa _\mathrm{tot}=10^9$ with $\kappa _\mathrm{tot}=\kappa _{0}+\kappa _{1}+\kappa _{2}=2\kappa +\kappa _{0}$ and $\kappa _{0}=\kappa /10$, $V_\mathrm{eff}=150\,\mu \mathrm{m}^3$, $\lambda = 1550$ nm, $P_\mathrm{in}=1$ fW, $n_0=1.4$, $n_2=3 \times 10^{-14}\,\mathrm{m^2/W}$^80^, and $\Omega =2.9 \times 10^4$ rad/s^53^.

### Quantum optical circulator

The routing behaviors of the circulator are shown in Fig. 1a,b and the port-to-port transmission spectra versus the detuning $\Delta$ are shown in Fig. 1c,d. When the frequency of the driving field is resonant with the CCW travelling mode, i.e. $\omega _L \approx \omega _\mathrm{ccw}= \omega _\mathrm{c}+\Delta _F$, the driving field transmitted with circulation in the direction $1\rightarrow 2 \rightarrow 3 \rightarrow 4\rightarrow 1$, see Fig. 1a. The circulation can be seen from the transmission spectra as shown in Fig. 1c,d. The condition of $\omega _L \approx \omega _\mathrm{ccw}$ is corresponding to the detuning $\Delta \approx \Delta _{F}=2.41\times 10^6$ rad/s. In this case, the driving field from ports 1 and 3 can not transmitted into the CW travelling mode for large frequency detuning, and also can not transmitted into the CCW travelling mode for the photon momentum with opposite direction, so that the driving fields from ports 1 and 3 transmit through the fibers directly to ports 2 and 4, respectively. In contrast, the driving fields from ports 2 and 4 couple to the CCW travelling mode resonantly with almost critical coupling, $\kappa _1=\kappa _2=\kappa \gg \kappa _0$, so that almost all the driving fields are injected into the CCW travelling mode and exported from ports 3 and 1, respectively. In addition, the circulator is not a perfect one with very little photon can transmit in the reversal direction. The isolation between the ports 1 and 2 (or 3 and 4) is about 16dB, and the isolation between the ports 2 and 3 (or 1 and 4) is about 18dB, around the frequency $\omega _L \approx \omega _\mathrm{ccw}$ (see green regions in Fig. 1c,d).

The circulator also can work in the reversal direction, i.e., $1\rightarrow 4 \rightarrow 3 \rightarrow 2\rightarrow 1$, at $\omega _L \approx \omega _\mathrm{cw}= \omega _\mathrm{c}-\Delta _F$, see Fig. 1b and the red regions of the transmission spectra in Fig. 1c,d. The routing behavior can be understood in a similar way as given above. As $\omega _L \approx \omega _\mathrm{cw}$, the driving field from ports 1 and 3 couple to the CW travelling mode resonantly with almost critical coupling, $\kappa _1=\kappa _2=\kappa \gg \kappa _0$, so that almost all the driving fields are injected into the CW travelling mode and exported from ports 4 and 2, respectively. In contrast, the driving fields from ports 2 and 4 can not transmitted into the CCW travelling mode for large frequency detuning, and also can not transmitted into the CW travelling mode for the photon momentum with opposite direction, so that the driving fields from ports 2 and 4 transmit through the fibers directly to ports 1 and 3, respectively. In addition, the circulator can work in the reversal direction by reverse rotating, i.e., the resonator spinning counter-clockwise (top view) at an angular velocity $\Omega$.

**Figure 2:**
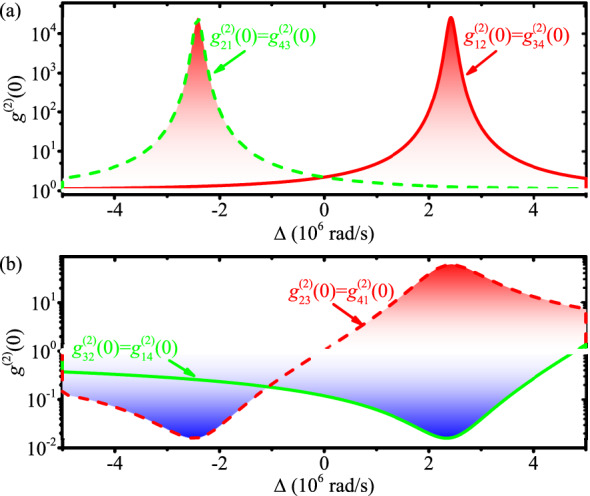
(Color online) The equal-time second-order correlation function of the port-to-port transmission photons versus the detuning $\Delta$.

Owing to the Kerr-type nonlinear interaction $U\approx 4.76 \times 10^6$ rad/s in the spinning resonator, the circulating photons exhibit quantum nonreciprocal routing behavior. The equal-time second-order correlation functions for the driving photons remaining in their original fiber (i.e., port $1 \leftrightarrows 2$ and $3 \leftrightarrows 4$) are shown in Fig. 2a, and all the transmitted photons show bunching effect. Specifically, when the arriving photons are large detuned from the CW or CCW mode in the spinning resonator, the transmitted photons show weak bunching effect with high transmittivity, i.e., $g^{(2)}_{21}(0)=g^{(2)}_{43}(0) \approx 1.26$ at $\Delta \approx 2.41\times 10^6$ rad/s and $g^{(2)}_{12}(0)=g^{(2)}_{34}(0)\approx 1.26$ at $\Delta \approx -2.41 \times 10^6$ rad/s. Instead, when the arriving photons are resonant with the CW or CCW mode in the spinning resonator, the transmitted photons show strong bunching effect with low transmittivity, i.e., $g^{(2)}_{21}(0)=g^{(2)}_{43}(0)\approx 2.5 \times 10^4$ at $\Delta \approx -2.41\times 10^6$ rad/s and $g^{(2)}_{12}(0)=g^{(2)}_{34}(0)\approx 2.5 \times 10^4$ at $\Delta \approx 2.41\times 10^6$ rad/s.

The equal-time second-order correlation functions for photons transferring between the two fibers, i.e., the transmission between ports $2 \leftrightarrows 3$ and $1 \leftrightarrows 4$, are shown in Fig. 2b. Clearly, the transmitted photons exhibit bunching or antibunching effect depending on the driving direction, i.e., nonreciprocal photon blockade^55^. With the detuning of $\Delta \approx 2.41\times 10^6$ rad/s, the photons transmitted from port 2 to 3 and from port 4 to 1 show strong antibunching effect. That is because the photons transmitted from port 2 to 3 and from port 4 to 1 go through the nonlinear CCW mode in the resonator, and the driving photons with the detuning of $\Delta \approx 2.41\times 10^6$ rad/s satisfy the the single-photon resonant condition $|\omega _L -\omega _\mathrm{ccw}| \ll \kappa _\mathrm{tot}$ and two-photon off-resonant condition $|2 \omega _L - 2\omega _\mathrm{ccw}- 2U| > \kappa _\mathrm{tot}$. In contrast, the photons transmitted from port 3 to 2 and from port 1 to 4 show strong bunching effect, because transmitted photons go through the nonlinear CW mode in the resonator, and satisfy the two-photon resonant driving $|2 \omega _L - 2\omega _\mathrm{cw}- 2U| \ll \kappa _\mathrm{tot}$ and single-photon off-resonant condition $|\omega _L -\omega _\mathrm{cw}| > \kappa _\mathrm{tot}$.

Moreover, with detuning of $\Delta \approx -2.41\times 10^6\,\mathrm{rad/s}$, all the transmitted photons exhibit antibunching effect. Nevertheless, the second-order correlation functions show different values for photons driving in different directions. The photons transmitted from port 3 to 2 and from port 1 to 4 show strong antibunching effect, because transmitted photons go through the nonlinear CW mode in the resonator, and satisfy the single-photon resonant condition $|\omega _L -\omega _\mathrm{cw}| \ll \kappa _\mathrm{tot}$ and two-photon off-resonant driving condition $|2 \omega _L - 2\omega _\mathrm{cw}- 2U| > \kappa _\mathrm{tot}$. Meanwhile, the photons transmitted from port 2 to 3 and from port 4 to 1 show much weaker antibunching effect, because transmitted photons go through the nonlinear CCW mode in the resonator, and satisfy conditions $|2 \omega _L - 2\omega _\mathrm{ccw}- 2U|> 2|\omega _L -\omega _\mathrm{ccw}| > \kappa _\mathrm{tot}$.

### The effect of back scattering

**Figure 3:**
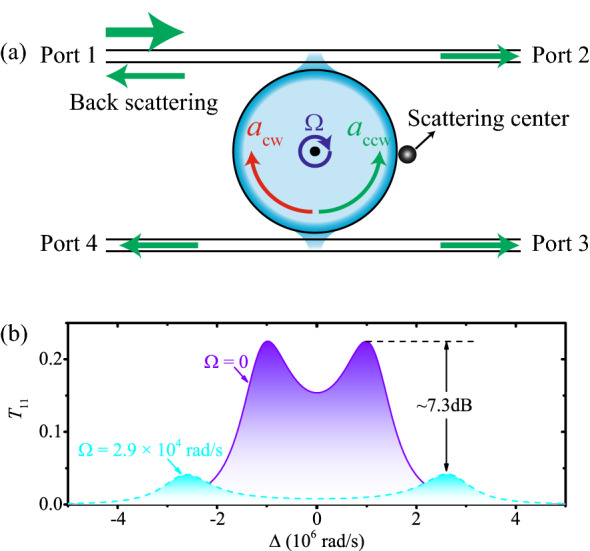
(Color online) (**a**) Schematic of the back scattering induced by a scattering center. (**b**) The back scattering spectra versus the detuning $\Delta$ with $J=2 \kappa$.

The CW and CCW travelling modes in the static resonator are degenerate with the same resonant frequency and field distributions. They couple to each other if the resonator deviates from its perfect azimuthal symmetry (surface roughness and material inhomogeneity), or an external scattering center as shown in Fig. 3a, which have been applied in nanoparticles detection^81^. Here, the coupling induces the back scattering of the input field and degrades the performance of the static circulator. So far we only consider the case without any back scattering. In this section, we will discuss the effect of back scattering on the performance of the circulator, and show that the quantum optical circulator here is robust against the back scattering. To be specific, we consider also the presence of a back scattering term $H_\mathrm{bs}$, with the intermodal coupling *J*,

11 $$\begin{aligned} H_\mathrm{bs}=J\left( a^{\dag }_{\mathrm {cw}}a_{\mathrm {ccw}}+a_{\mathrm {ccw}}^{\dag }a_{\mathrm {cw}}\right) , \end{aligned}$$

in the total Hamiltonian given in Eq. (2). Without loss of generality, we will only show the results for photons driving from port 1, i.e., $T_{11}$, by solving the master equation numerically.

**Figure 4:**
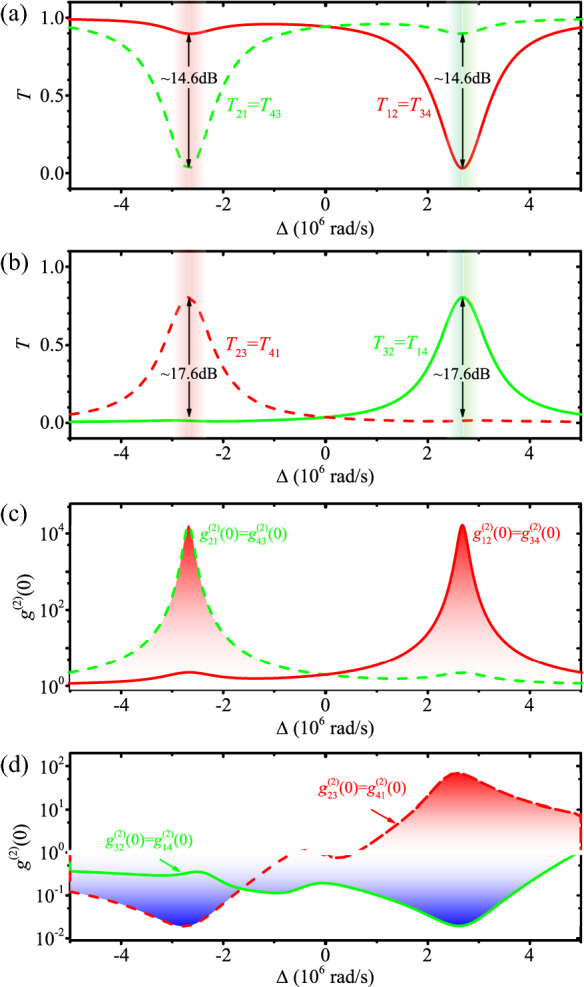
(Color online) (**a**) and (**b**) the port-to-port transmission spectra versus the detuning $\Delta$ with back scattering of $J=2\kappa$; (**c**) and (**d**) the equal-time second-order correlation function of the port-to-port transmission photons versus the detuning $\Delta$ with back scattering of $J=2\kappa$.

The back transmission coefficient $T_{11}$ for a spinning Kerr resonator with angular velocity $\Omega =2.9 \times 10^4$ rad/s is given in Fig. 3b under strong back scattering $J=2\kappa$. The back scattering effect is given with $T_{11}\approx 4.2\%$, which is relatively small with strong back scattering. In order to make this clearer, we also show the back transmission coefficient $T_{11}$ for a microtoroid resonator without spinning, i.e., $\Omega =0$ in Fig. 3b. In this case, the maximum value of $T_{11}$ reaches $22.5\%$. Clearly, the spinning resonator is more robust against back scattering than that in a static device, since the back scattering effect is significantly suppressed for about 7.3 dB (depending on *J*), as shown in Fig. 3b. This advantage can be understood with fact that both the frequency and momentum are shifted by rotation, so that both the frequency and momentum matching conditions between CW and CCW modes can not satisfied in the spinning resonator when the scattering centers reverse the direction of the photon momentum.

The transmission spectra and equal-time second-order correlation functions, with strong back scattering $J=2\kappa$, are shown in Fig. 4. These results show the effect of back scattering in two ways. First, the isolation between the ports 1 and 2 (or 3 and 4) decreases from 16 to 14.6 dB, and the isolation between the ports 2 and 3 (or 1 and 4) decreases from 18 to 17.6 dB. Second, the frequency for maximal isolation is shifted from $\Delta \approx \pm \, 2.41 \times 10^6$ rad/s to $\pm \, 2.68 \times 10^6$ rad/s due to the energy repulsion of the CW and CCW travelling modes induced by intermodal coupling. Clearly, except for the slight frequency shift in the transmission spectra, quantum correlations of the transmitted photons do not change much, i.e., the circulator still exhibits quantum nonreciprocal routing behavior even for strong back scattering ($J=2\kappa$). These results confirm that, when operating as either an optical isolator or a circulator, the spinning device is robust even in a perturbed environment with strong back scattering effects. We remark that this of course does not mean that the spinning device can not serve also as a highly-sensitive sensor of particles. In fact, this possibility was already revealed in a very recent work^54^ which indeed observed a larger optical mode splitting induced by the nanoparticles near a spinning resonator (see Ref.^54^ for more details).

## Discussion

In summary, we have proposed a scheme to realize a four-port quantum optical circulator for critical coupling of a spinning Kerr resonator to two tapered fibers. The routing direction of the circulator depends on the frequency of the incident photons and the rotating direction of the resonator. The quantum correlations of the transmitted photons also are direction dependent: the photons remaining in their original fibers exhibit bunching effect with direction dependent bunching strength, and nonreciprocal photon blockade occurs for photons transferred between the two fibers. Thus, the quantum optical circulator based on a spinning Kerr resonator can be used for routing and processing of both classical and quantum signals. Furthermore, we discussed the back scattering effect induced by the intermodal coupling between counter-travelling optical modes, and showed that the quantum optical circulator for a spinning Kerr resonator is much more robust against the back scattering by comparing the results for a microtoroid resonator with and without spinning. Beyond that, in contrast to the nonreciprocal devices with two ports, the four-port quantum optical circulator enables us to build two- and three-dimensional networks for implementing photonic quantum simulation^82^.

## Methods

In order to obtain the transmission and statistical properties of the photons quantitatively, we numerically study the dynamics of the system by introducing the density operator $\rho$ and solving the master equation^83^

12 $$\begin{aligned} \frac{\partial \rho }{\partial t} =-i\left[ H_{\mathrm {sys}},\rho \right] +\kappa _\mathrm{tot} L[a_{\mathrm {cw}}]\rho +\kappa _\mathrm{tot} L[a_{\mathrm {ccw}}]\rho , \end{aligned}$$

where $L[o]\rho =o\rho o^{\dag }-\left( o^{\dag }o\rho +\rho o^{\dag }o\right) /2$ denotes a Lindbland term for an operator *o*; $\kappa _\mathrm{tot}=\kappa _{0}+\kappa _{1}+\kappa _{2}$ is the total decay rate.
